# Correspondence: Reply to ‘Space-time asymmetry undermines water yield assessment'

**DOI:** 10.1038/ncomms11604

**Published:** 2016-05-12

**Authors:** Xiuzhi Chen, Xiaohua Wei, Ge Sun, Ping Zhou, Guoyi Zhou

**Affiliations:** 1 South China Botanical Garden, Chinese Academy of Sciences, Guangzhou 510650, China; 2 Earth & Environmental Sciences and Physical Geography, University of British Columbia (Okanagan campus), Kelowna, British Columbia, Canada V1V1V7; 3 Eastern Forest Environment Threat Assessment Center, USDA Forest Service, Raleigh, North Carolina 27606, USA; 4 Department of Forest Ecology, Guangdong Academy of Forestry, Guangzhou 510520, China

We appreciate the comments by Berghuijs and Woods^1^ on our study^2^ that investigates how land cover and climate affect watershed water yield at the global scale. We used published data worldwide to examine the validity of Fuh's equation and address the interactions of water yield coefficient (*R/P*) with wetness index (*P*/*PET*) and watershed characteristics (*m*).

Berghuijs and Woods^1^ argue that we implicitly assumed that there is symmetry between spatial and temporal partitioning of precipitation into streamflow and evaporation. We, however, disagree. Fuh and other Budyko frameworks are based on energy and climate under a steady state^3^,^4^. If a system is at steady state (for example, change in water storage is zero), the equations apply. It can be applied to different watersheds (spatial scale) and a single watershed over different intervals (temporal scales; again each interval must meet the steady-state condition). More importantly, Fuh's is a theoretical framework. Thus, when it applies to a real-life data set, asymmetry from real-life data is likely expected. The difference identified (27.9%) by Berghuijs and Woods^1^ can be attributed to various sources, including the quality of the data set, non-stationarity of time-series data (as caused by climate change) and the inconsistent application of methods (in Berghuijs and Woods^1^, the first method is based on the theoretical framework while the second is a simplified linear regression without any physical basis). Application of an empirical linear equation by Berghuijs and Woods^1^ is problematic, as evidenced by impossible negative *α* values in their Fig. 1 (ref. 1). In addition, *α* value in Berghuijs and Woods^1^ (equivalent to *∂R/P/∂P/PET*) is a regressed slope of their equation (2) based on long-term observations in a watershed. As a result, a watershed has only one *α* value. How does it reflect temporal change? In fact, no temporal sensitivity is shown by Berghuijs and Woods^1^. Thus, we do not believe that they provide sufficiently robust evidence to support their claims regarding space-time asymmetry.

Berghuijs and Woods^1^ state that *m* should include the effects of climate intra-annual variability (for example, precipitation seasonality). As described above, Fuh's equation describes the climate and energy balances in a steady watershed. Thus, climate intra-annual variability should not be involved. Because climate is already represented in Fuh's equation, *m* is considered by many authors^5^ as a parameter for representing watershed properties or characteristics. However, Berghuijs and Woods^1^ do raise an important question regarding possible interactions between climate and *m*, which we feel is an open question for future research.

The two critical values shown in Fig. 1b and Fig. 1c of Zhou *et al*.^2^ define the boundaries of the sensitivities of *R*/*P* to both *P*/*PET* and *m* values. They classify the possible combinations of variables *P*/*PET* (0, ∞) and *m* (1, ∞) into four different ranges in which the sensitivities of *R*/*P* to both *P*/*PET* and *m* are varied (Zhou *et al*.^2^, Table 1). Berghuijs and Woods^1^ state that comparisons of those two sensitivities are not meaningful for assessing water yield changes (Δ*R*/*P*) unless they are combined with typical changes of climate (Δ*P*/*PET*) and watershed parameter (Δ*m*). We agree that comparisons can be more meaningful if those two sensitivities are integrated with those typical changes for assessing water yield changes. However, our paper^2^ attempts to validate whether Fuh's equation is an appropriate framework for assessing the effects of climate and land cover on watershed hydrology using the global published data. Our evaluation is thus within a more theoretical context. With our theoretical analysis, we were able to quantify the sensitivities and relative importance of climate (*P*/*PET*) and *m* to water yield changes, which we feel is meaningful and important. We do, however, agree that more testing on critical values and their sensitivities should be done with field data in the future.

Berghuijs and Woods^1^ further questions the extent to which Zhou *et al*.^2^ provided an explanation for cases where water yields from forests can potentially exceed those from grasslands. Zhou *et al*.^2^ show that the sensitivity of *R*/*P* to *m* is very low in the situations of *P*/*PET*>>1 and *m*>>2 (Zhou *et al*.^2^, Fig. 1b,c), and, under those conditions, any land cover change (for example, afforestation or deforestation) may play a limited role in influencing *R*/*P*. Therefore, if afforestation cools local land surface temperature^6^,^7^ and consequently increases *P*/*PET*, then afforestation can potentially increase water yield. This conclusion is based on Fuh's theoretical equation, not on speculation.

In summary, we feel that the claim of ‘space-time asymmetry' by Berghuijs and Woods^1^ lacks sufficient evidence to undermine the conclusions of Zhou *et al*.^2^, and that some claims may be the result of a mis-interpretation of our original study.

## Additional information

**How to cite this article:** Chen, X. *et al*. Correspondence: Reply to ‘Space-time asymmetry undermines water yield assessment'. *Nat. Commun.* 7:11604 doi: 10.1038/ncomms11604 (2016).
